# The Use of an Early Alert System to Improve Compliance with Sepsis Bundles and to Assess Impact on Mortality

**DOI:** 10.1155/2012/980369

**Published:** 2012-02-26

**Authors:** Jennifer Anne LaRosa, Noeen Ahmad, Monica Feinberg, Monica Shah, Roseann DiBrienza, Sean Studer

**Affiliations:** Newark Beth Israel Medical Center, Newark, NJ 07112, USA

## Abstract

*Introduction*. Diagnostic and therapeutic guidelines, organized as sepsis bundles, have been shown to improve mortality, but timely and consistent implementation of these can be challenging. Our study examined the use of a screening tool and an early alert system to improve bundle compliance and mortality. *Methods*. A screening tool was used to identify patients with severe sepsis or septic shock and an overhead alert system known as Code SMART (Sepsis Management Alert Response Team) was activated at the physician's discretion. Data was collected for 6 months and compliance with bundle completion and mortality were compared between the Code SMART and non-Code SMART groups. *Results.* Fifty eight patients were enrolled −34 Code SMART and 24 non-Code SMART. The Code SMART group achieved greater compliance with timely antibiotic administration (*P* < 0.001), lactate draw (*P* < 0.001), and steroid use (*P* = 0.02). Raw survival and survival adjusted for age, leucopenia, and severity of illness scores, were greater in the Code SMART group (*P* < 0.05, *P* = 0.03, and *P* = 0.01). *Conclusions*. A screening tool and an alert system can improve compliance with sepsis bundle elements and improve survival from severe sepsis and septic shock.

## 1. Introduction

Sepsis is one of the leading causes of mortality worldwide. Over 750,000 hospitalizations occur in the United States each year, with approximately 200,000 deaths [1]. Because of such high mortality, guidelines known as Early Goal Directed Therapy (EGDT) were developed to increase sepsis awareness and direct therapy, thereby reducing mortality [2]. The Surviving Sepsis Campaign subsequently classified EGDT elements into a sepsis resuscitation bundle and a sepsis management bundle [2]. These bundles direct specific diagnostic and treatment strategies within designated time frames. Better outcomes have been demonstrated when compliance with bundle elements is high [3]. 

Consistent implementation of sepsis bundles has been challenging. Noncompliance with sepsis bundles has been demonstrated to increase in-hospital mortality for septic patients, while compliance with the resuscitation bundle, even if extended from the recommended time frame, decreases mortality [4, 5]. We sought to develop an easy to implement, low-cost system that would increase bundle compliance and improve survival. This was initially developed as a quality improvement initiative and ultimately became a standard of care in our facility. We hypothesized that the use of an early alert system that brought an interdisciplinary team rapidly to the patient's bedside would increase compliance with bundle elements and improve survival for those presenting with severe sepsis or septic shock.

## 2. Materials and Methods

### 2.1. Design, Overview, and Setting

This prospective cohort study was performed in an Intensive Care Unit (ICU) in a tertiary care, urban teaching hospital of 673 beds. This ICU admits adult medical and surgical patients with the exception of patients who have undergone cardiothoracic surgery. Our study protocol was reviewed and approved by our Institutional Review Board (IRB) with a waiver of informed consent as the study was deemed a performance improvement intervention. The primary investigator supervised collection of study data during each patient's ICU admission. 

### 2.2. Patients

All adult patients admitted to the ICU from April 7th, 2009 through October 6th, 2009 were screened for study entry. Patients with severe sepsis or septic shock at the time of ICU admission (patients who developed severe sepsis or septic shock while in the ICU were not included), as defined by Surviving Sepsis Campaign criteria [2], were identified through the use of a written screening tool based on standard definitions of these disorders [5]. This tool was utilized by Emergency Department (ED) and Internal Medicine physicians to identify potential participants. (Table 7—screening tool). All patients who met criteria for severe sepsis or septic shock and all those on whom a Code SMART was called were admitted to the ICU. Additionally, we reviewed hospital admissions for the ICD-9 codes corresponding to sepsis, septic shock, and undifferentiated shock to ensure that no patients during the study period admitted to the “general ward” or other ICUs in our institution.

### 2.3. Early Alert System Protocol

**Figure 1:**
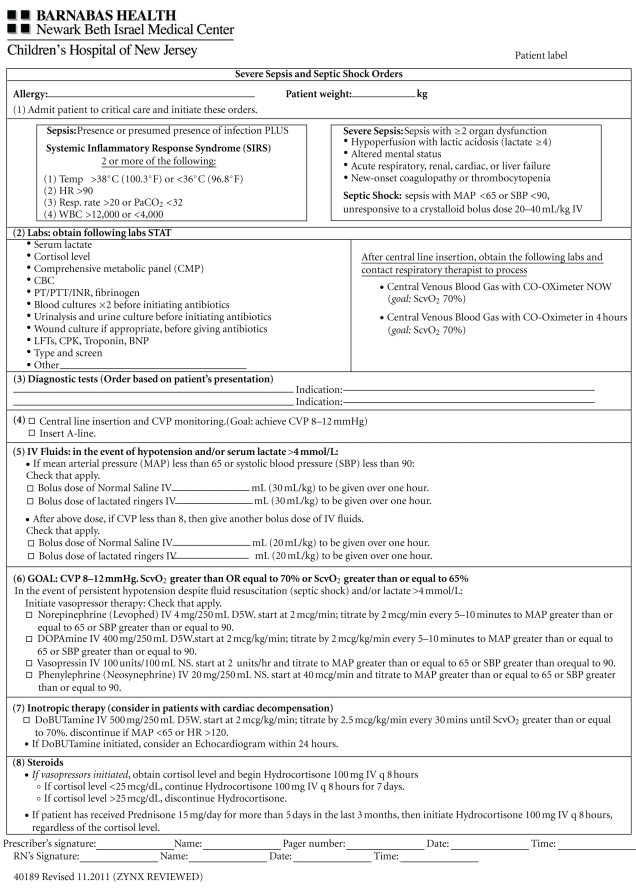

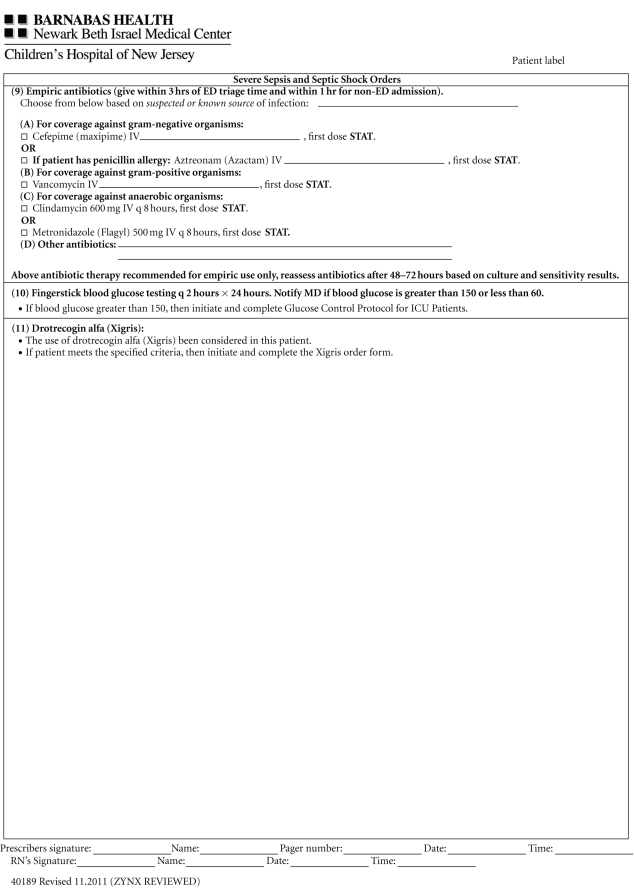


ED and ICU physicians, nurses, respiratory care practitioner, and pharmacists were educated regarding EGDT and sepsis bundles through lectures and bedside teaching. Following these educational sessions, a written screening tool was provided to staff to facilitate the identification of appropriate patients. Patients presenting to the ED or who demonstrated clinical deterioration while admitted to the medical or surgical wards, who met two or more criteria on the screening tool (thereby demonstrating that they were likely to have severe sepsis or septic shock) were intended to trigger an overhead alert known as Code SMART (SMART = Sepsis Management Alert Response Team). Such a trigger occurred based on initial triage evaluation and took place within 30 minutes of the patient's arrival to the ED. Responders included an (ICU) physician, (pulmonary and critical care medicine fellow or attending physician), ICU nurse, respiratory care practitioner, and pharmacist. Sepsis resuscitation and management bundle elements were implemented upon the Code SMART team's arrival at the bedside within 10 minutes of the overhead alert call. This was accomplished using a standardized order set to assess necessary diagnostic and treatment information (see Figure 1—order set). Upon arrival to the ICU, all patients with severe sepsis or septic shock were managed according to a standardized protocol also based on bundle elements (see Table 8—progress note). Patients admitted with severe sepsis or septic shock, on whom a Code SMART was not triggered, were then managed by the same protocol (order set and progress note). However, for Non-Code SMART patients, management was at the discretion of the treating physician prior to ICU arrival.

### 2.4. Data Documentation

For all patients included in this study, the following demographic data was collected via the electronic medical record: age, gender, and ethnicity. Severity of illness was assessed in all patients using MED scores [6–8]. The origin of the admission to the ICU for each patient was recorded (ED versus general medical-surgical ward). Potential differences in the severity of illness between the two groups were further assessed by evaluating the following parameters: (1) site of sepsis, (2) rates of positive blood and urine cultures, (3) the numbers of organs involved, (4) and the presence or absence of shock.

The primary endpoint was compliance with each of the ten individual resuscitation and management bundle elements. A written case report form was completed by the Code SMART team leader at the time of the Code SMART and in the ensuing 24 hours. The secondary endpoint was mortality which was captured by the team leader at the time of the patient's discharge.

### 2.5. Statistical Analysis

Age, gender, ethnicity, and severity of illness as measured by MED scores were compared between the participants who were in the Code SMART arm versus those who were not part of Code SMART. An independent samples *t*-test was used to determine if age and severity of illness had a relationship with intervention arm, while a chi-square analysis was used for gender and ethnicity. Independent samples *t*-tests were also used to determine any statistically significant differences with respect to each individual bundle element and survival at discharge and intervention arm (Code SMART versus Non-Code SMART). Finally, a logistic regression analysis was performed on mortality as the outcome and the predictors of intervention group (Code SMART versus Non-Code SMART), MED scores, and leucopenia. A second logistic regression analysis was performed by adding age to the first model. All analyses were performed using SPSS.

## 3. Results

Of 447 ICU admissions during the study period, 58 (7.7%) were admitted with a primary diagnosis of severe sepsis or septic shock and all were included in the study. Fifty-one of these patients were admitted to the hospital from the ED and of these, 32 triggered a Code SMART. The remaining 7 patients were admitted from general medical-surgical wards, of which 2 were managed with a Code SMART.

As shown in Table 1, the two groups were similar demographically in terms of gender (*χ* ^2^(1, *N* = 58) = .68, *P* = .41), ethnicity (*χ* ^2^(3, *N* = 58) = 2.23, *P* = .53), and MED scores (*P* = .32). However, there was a statistically significant difference in age between the two groups, with the Code SMART patients significantly being older than the Non-Code SMART patients. The mean ages were 70 (SD = 2.3) and 61 (SD = 3.4) for the Code SMART and Non-Code SMART patients, respectively. Furthermore, there were no statistically significant differences between the two groups with respect to percentage of positive cultures, source of sepsis, presence of absence of shock, and when zero, one, or more than four organs were involved with the sepsis. There were statistically more patients in the Code SMART group with two or three organs involved in the disease process.

Ten sepsis bundles were compared collectively and individually between the two groups. Collectively, compliance with sepsis bundles was achieved more frequently in the Code SMART group (*P* = .01; Table 2).  Table 3 outlines the specific elements that reached statistical significance with a greater compliance in the Code SMART population including the administration of antibiotics (*P* < .001), lactate blood draw (*P* < .001), and the use or documentation of the consideration of steroid use. (*P* = .02) Blood culture draws (*P* = .07) and intravenous fluid administration (*P* = .11) trended towards greater compliance in the Code SMART group but did not reach statistical significance.

Survival at discharge was compared between the two study groups and was higher in the Code SMART group; this difference achieved statistical significance (*P* = .04; see Table 4). When this data was adjusted for the use of Code SMART, MED scores, and leucopenia, the Code SMART patients enjoyed a sevenfold reduction in their risk of mortality (Table 5). When mortality calculations were further adjusted for these factors plus age, the survival benefit was increased by a factor of greater than 32 (Table 6).

## 4. Discussion

This study demonstrates that an early alert system is an effective tool for increasing compliance with sepsis resuscitation and management bundle elements as its primary outcome. Of the 10 bundle elements, the use of antibiotics, lactate blood draw, and the use or consideration of steroids were completed more consistently in the Code SMART group compared to those treated without this alert system. The most remarkable of these findings was the statistically significant difference between antibiotic administration timing in the two groups, strongly favoring timely administration in the Code SMART group. It appears that this difference is not attributable to diagnostic uncertainty but due to failure to utilize Code SMART.

 Inspiratory plateau pressures <30 cm H_2_O were achieved equally well in both groups. Although the final seven elements did not achieve statistical significance, there was a trend towards better compliance for six of the elements in the Code SMART group. In addition, a significant reduction in unadjusted mortality in the Code SMART patients was observed. After adjusting for leucopenia (an independent risk factor for mortality from sepsis [9]) and MED scores, the mortality benefit in the Code SMART becomes more significant. Additional adjustment for age (another independent risk factor for sepsis mortality [10]), further implies this survival benefit. The increased number of organs involved in the sepsis process in the Code SMART group would tend to bias against this group's better overall survival, which makes the observed mortality benefit in the Code SMART cohort a more robust finding.

Earlier antibiotic usage within the designated time frame was the most statistically significant in this study for the Code SMART group. Not only has a combination of two antibiotic therapies in the initial treatment of septic shock shown to reduce mortality, but time of triage to antibiotic administration has also been proven to decrease mortality [11–15]. Animal models have shown that a delay in antibiotic administration following the onset of hypotension is associated with an increase in inflammatory mediators [16]. Prompt treatment is key, and reducing antimicrobial burden by early antibiotic administration not only decreases mortality in septic shock, but also decreases pressor/inotrope free days, and ventilator days [12].

Increased lactate results from tissue hypoperfusion. Decreased perfusion to tissue affects mitochondrial oxidative phosphorylation thereby shifting energy metabolism to anaerobic glycolysis and the production of serum lactic acid [17]. Measuring lactate levels may alert providers to perfusion abnormalities that prompt therapeutic changes that improve outcome. Therefore, interventions such as Code SMART that improve the measurement of lactate may facilitate these goal-directed interventions.

Early Goal-Directed Therapy and the Surviving Sepsis Campaign demonstrated that early recognition and management of sepsis could save lives through implementation of ten bundle elements. In addition, early alert system such as Code STEMI for patients with ST-segment elevation myocardial infarctions have demonstrated an improved rate of survival with early recognition and implementation of appropriate therapy. Our study demonstrates that such an early intervention can be useful in severe sepsis and septic shock as well in that it increases compliance with bundle elements and improves survival.

A few potential limitations of this study deserve mention. The first limitation is our small sample size obtained over a six-month study period in a single urban academic medical center. As a follow-up study, a larger sample size from multiple centers would be desirable to determine whether the observed positive trends in seven of the ten bundle elements would reach statistical significance. This data did not include patients with cardiothoracic surgery and subsequent severe sepsis or septic shock during the same hospitalization; therefore, these results may not be applicable to this group. A final limitation was that Code SMART was not triggered in all cases that met screening criteria during this first six months of the use of this tool. Potential barriers to triggering a Code SMART in all patient may be the same as those faced with regard to compliance to EGDT in the ED. Since Code SMART has become established, it has become a standard of care in our facility to call a Code SMART for every patient meeting screening criteria.

In conclusion, severe sepsis and septic shock are medical emergencies with an extremely high mortality rate. Code SMART was shown to improve sepsis bundle compliance in our institution as well as raw and adjusted mortality. Code SMART functions as an early alert system that is easy to implement, and essentially without cost, making it an attractive tool for institutions to consider when meeting the goals of early goal-directed therapy for severe sepsis and septic shock.

## Figures and Tables

**Table 1:** Patient characteristics between Code SMART and Non-Code SMART Groups.

	Code Smart	Non-Code Smart	*P*
	*N* = 34	*N* = 24	
Mean (SD) Age (years)	70 (2.26)	61 (3.41)	.03
Ethnicity			
Caucasian	1 (3%)	3 (13%)	.16
African American	28 (82%)	17 (71%)	.31
Hispanic	3 (9%)	2 (8%)	.95
Other	2 (6%)	2 (8%)	.73
Gender (females)	15 (44%)	16 (67%)	.42
Mean (SD) MEDS	11.44 (.94)	9.96 (1.13)	.32
Sepsis			
Urinary tract infection	10 (29%)	7 (29%)	.80
Pneumonia	18 (53%)	8 (33%)	.25
Cultures-positive			
Blood	13 (38%)	8 (33%)	.96
Urine	16 (47%)	7 (29%)	.26
Shock present	25 (74%)	15 (63%)	.38
Number of organs involved			
0	2 (6%)	2 (8%)	.72
1	8 (24%)	5 (21%)	.81
2	8 (24%)	14 (50%)	.01
3	10 (29%)	2 (8%)	.05
4+	6 (18%)	1 (4%)	.13
Leucopenia	3 (9%)	2 (8%)	.95

**Table 2:** Difference of means test for compliance with sepsis bundles.

	Observed	Mean (SD)
Code SMART	34	6.50 (1.98)
Non-Code SMART	24	5.21 (2.28)
		*t* = 2.7*

*P = 0.1.

**Table 3:** Compliance with individual sepsis tasks.

	Code SMART proportion (S.D.)/*n*	Non-Code SMART proportion (S.D.)/*n*	*t* score	*P* value
Antibiotics	.91 (.03)/34	.54 (.10)/24	−4.57	**<.001**
Blood cultures	.91 (.03)/34	.83 (.08)/24	−1.86	0.07
IV Fluids	0.71 (.08)/34	.50 (.10)/24	−1.59	0.11
Lactate	.94 (.04)/34	.63.10/24	−3.24	**<.001**
CVP	1 (0)/18	.89 (.11)/9	−1.44	0.16
MV02	.50 (.11)/22	.38 (.18)/8	−0.59	0.56
Steroids	.55 (.08)/34	.25 (.09)/24	−2.41	**0.02**
APC	.61 (.08)/34	.41 (.10)/24	−1.52	0.14
Glucose	.62 (.08)/34	.71 (.09)/24	0.71	0.48
IPP	1 (0)/7	1 (0)/12	n/a	n/a

**Table 4:** Survival at discharge—Code SMART versus Non-Code SMART.

	Total patients	Patients alive at discharge
Code SMART	34	31 (91%)
Non-Code SMART	24	17 (71%)
		*t* = −0.2.06

*P < .05.

**Table 5:** Model 1—logistic regression of mortality controlling for +/− use of Code SMART, MEDS, and leucopenia.

	Model 1	*P* value	Odds ratios
Code SMART	2.11 (.96)	**0.03**	**7.34**
MEDS	−.15 (.08)	0.06	0.83
Leucopenia	−2.00 (1.35)	0.14	0.11
Constant	2.63 (.99)	0.01	—
*N*	58		
*χ* ^2^	13.81	0.00	

**Table 6:** Model 2—Logistic regression of mortality controlling for +/− use of Code SMART, MEDS, leucopenia, and age.

	Model 2	*P* value	Odds ratios
Code SMART	3.492635 (1.345015)	**0.01**	**32.88**
MEDS	−.2171075 (.1181574)	0.07	0.80
Leucopenia	−2.628451 (1.594963)	0.10	0.07
Age	−.1090305 (.0494949)	0.03	0.89
Constant	10.68133 (5.680985)	0.06	—
*N*	58		
*χ* ^2^	23.94	0.00	

**Table 7:** Evaluation for sepsis—screening Tool.

**Instructions:** Use this screening tool to screen patients for sepsis.
(1) Is the patient's history suggestive of a new infection:
□ no
□ yes, if yes suspected source
□ Pneumonia, empyema
□ Urinary tract infection
□ Acute abdominal infection
□ Meningitis
□ Skin/soft tissue infection
□ Bone/joint infection
□ Wound infection
□ Bloodstream catheter infection
□ Endocarditis
□ Implantable device
□ Other
(2) Are any two signs and symptoms of infection present AND new to the patient?
□ Hyperthermia (>101°F or 38.3°C)
□ Hypothermia (<96.8°F or 36°C)
□ Tachycardia (>90 bpm)
□ Tachypnea (>20 bpm)
□ Acutely altered mental status
□ Leukocytosis (WBC count >12,000)
□ Leukopenia (WBC count <4,000)
□ >10% immature neutrophils
*If* the answer is yes to both questions 1 and 2, *suspicion of infection* is present:
□ Obtain: serum lactate, blood cultures, CBC with diff, basic chemistry labs, bilirubin
□ Pertinent diagnostic tests ___________________________________
(3) Are any of the following organ dysfunction criteria present AND acute:
□ SBP < 90 mmHg or MAP < 65 mmHg
□ SBP decrease >40 mmHg from baseline
□ Bilateral pulmonary infiltrates with a new (or increased) oxygen requirement to maintain SpO_2_ > 90%
□ Creatinine > 2 mg/dL or Urine Output < 0.5 mL/kg/hr for more than 2 hours
□ Bilirubin > 2 mg/dL
□ Platelet count <100,000
□ Coagulopathy (INR > 1.5 or aPTT > 60 secs)
□ Serum lactate > 2 mmol/L
*➢*If *suspicion of infection* is present AND *organ dysfunction* is present, the patient meets criteria for *Severe sepsis*.
*➢*If patient meets *Severe sepsis* criteria, AND has refractory hypotension (>60 min AND/OR unresponsive to fluid bolus of 20 mL/kg), the patient meets criteria for *Septic shock*.
**Initiate and complete the severe sepsis protocol.**

**Table 8:** Sepsis patient daily progress note.

*Date of Admission*:_____________________________ *Admitting Diagnosis*:______________________________________________
*Sepsis Information*

Was sepsis present on admission (including ED stay)?: yes no
If no, date/time noted to be present: _________________________________________________________________________________________________________
Source of sepsis:______________________________________________________________________________________________________________________________
Evidence of infection (CXR, U/A, etc.): ___________________________________________________________________________________________________
Positive blood cultures and date: ________________________________________________________________________________________________________
End-organ damage:___________________________________________________________________________________________________________________________
*Antibiotic and Other Treatment*

Initial antibiotic therapy:____________________________________________________________________________________________________________________
Current antibiotic therapy and start date:__________________________________________________________________________________________________
Anticipated duration of antibiotic therapy: _________________________________________________________________________________________________
Route of administration and need for long-term access: ___________________________________________________________________________________
Steroids considered/ Why given or not: _____________________________________________________________________________________________________
Xigris considered/Why given or not:________________________________________________________________________________________________________
Please provide a brief summary of why the patient needs to remain in the hospital and cannot receive further treatment as an outpatient.
____________________________________________________________________________________________________________________________________________________
____________________________________________________________________________________________________________________________________________________
____________________________________________________________________________________________________________________________________________________
Signature/Title Date /Time
